# Characterization of multiple myeloma osseous lesions and diffuse infiltration pattern by 18F-FDG-PET/CT, static MRI and diffusion-weighted MR Imaging (DWI-MRI): a comparative multimodality imaging study

**DOI:** 10.1186/1470-7330-14-S1-P33

**Published:** 2014-10-09

**Authors:** J Mosebach, C Sachpekidis, N Fard, T Wilhelm, M Wilhelm, J Hillengass, A Dimitrakopoulou-Strauss, HP Schlemmer, S Delorme

**Affiliations:** 1 Department of Radiology, German Cancer Research Center, Heidelberg, Germany; 2 Clinical Cooperation Unit Nuclear Medicine, German Cancer Research Center, Heidelberg, Germany; 3 Department of Radiology, University of Freiburg, Freiburg, Germany; 4 Department of Medicine V, Multiple Myeloma Section, University of Heidelberg, Heidelberg, Germany

## Aim

To compare the detection rates of different imaging modalities for nodular bone marrow (BM) infiltrates and osteolyses in patients with multiple myeloma (MM).

## Methods

55 Patients diagnosed according to the International Myeloma Working Group criteria (2003) were referred to consecutive imaging diagnostics including 18F-FDG PET/CT, T1-weighted and short-tau inversion recovery (STIR) whole-body MRI (WB-MRI) as well as diffusion weighted MRI (DWI). Images were reviewed and matched on a lesion-by-lesion basis for the location and number of focal osseous MM lesions by two radiologists and one nuclear medicine physician, grading the conspicuity of each lesion in CT, T1w, T2 STIR, DWI and ADC-Map separately. PET/CT data were compared based on qualitative and semi-quantitative (SUV) evaluation. A scoring system was used, with a certainty score as the reference for each lesion resulting from an evaluation considering all imaging modalities simultaneously. The analysis was carried out without knowledge of the previous treatment or the stage of disease.

## Results

In static MRI and DWI different infiltration patterns were observed and PET/CT revealed comparable patterns of tracer uptake: negative, focal, diffuse and mixed. MRI including DWI demonstrates both active as well as inactive MM lesions and displays numerous additional PET-negative lesions.

## Conclusion

Possible reasons for lesions being PET positive or negative may be their activity or cellular density of focal BM infiltrates. Additionally, nodules may be masked by surrounding, diffuse BM infiltration. To this date, PET/CT and DWI/MRI are complementary methods, as both modalities interrogate different biophysical tissue properties (cellular density vs glucose metabolism).

